# Ablative radiation therapy for locally advanced pancreatic cancer: techniques and results

**DOI:** 10.1186/s13014-019-1309-x

**Published:** 2019-06-06

**Authors:** Marsha Reyngold, Parag Parikh, Christopher H. Crane

**Affiliations:** 1 Department of Radiation Oncology, Memorial Sloan Kettering Cancer Center, 1275 York Ave, New York, NY 10065 USA; 2 Department of Radiation Oncology, Henry Ford Cancer Institute, 2799 W Grand Blvd, Detroit, MI 48202 USA; 3 Department of Radiation Oncology, Memorial Sloan Kettering Cancer Center, 300 East 65th St. Rm1509, New York, NY 10065 USA

**Keywords:** Pancreatic adenocarcinoma, Ablative radiation, Hypofractionated ablative radiation, IGRT, CBCT guided radiation therapy, MRI guided radiation therapy

## Abstract

Standard doses of conventionally fractionated radiation have had minimal to no impact on the survival duration of patients with locally advanced unresectable pancreatic cancer (LAPC). The use of low-dose stereotactic body radiation (SBRT) in 3- to 5-fractionshas thus far produced a modest improvement in median survival with minimal toxicity and shorter duration of treatment, but failed to produce a meaningful difference at 2 years and beyond. A much higher biologically effective dose (BED) is likely needed to achieve tumor ablation The challenge is the delivery of ablative doses near the very sensitive gastrointestinal tract. Advanced organ motion management, image guidance, and adaptive planning techniques enable delivery of ablative doses of radiation (> = 100Gy BED) when more protracted hypofractionated regimens or advanced image guidance and adaptive planning are used. This approach has resulted in encouraging improvements in survival in several studies. This review will summarize the evolution of the radiation technique over time from conventional to ablative and describe the practical aspects of delivering ablative doses near the GI tract using cone beam CT image (CBCT) guidance and online adaptive MRI guidance.

## Background

Unresectable locally advanced pancreatic cancer continues to carry a grim prognosis with a median survival of 10–16 months even in the context of the significant improvement in chemotherapy options seen in the last two decades [1]. At least a third of the patients die of complications related to local progression with or without any evidence of metastatic disease [2], and local progression may predominate as the cause of death for patients surviving more than 15 months regardless of their metastatic status [2, 3]. This, underscores the importance of local control and suggests that improved local control can translate into improved survival, at least for a subset of patients. As a local modality, radiation therapy has been extensively tested in this setting.

## Lessons from conventional radiotherapy experience

Standard radiotherapy options, commonly delivering 40 to 60 Gy in 1.8–2.0 Gy per fraction add minimal to no survival benefit for patients with locally advanced unresectable pancreatic cancer (LAPC) who have received chemotherapy. These doses were based on the tolerability of large field radiation to the stomach and duodenum in the 2D and 3-D era, and have been shown to provide a modest local tumor control benefit only. Five phase III randomized trials evaluated the role of standard doses of radiation delivered with concurrent chemotherapy to chemotherapy alone in the treatment of locally advanced pancreatic cancer with mixed results [4–8]. Inconsistent results were seen ven when considering the three trials performed in the 2000s in patients receiving (neo)adjuvant gemcitabine (Table 1) [5, 7, 8]. The Fédération Francophone de Cancérologie Digestive and Société Française de Radiothérapie Oncologique (FFCD-SFRO) randomized 119 patients to chemoradiation with 60Gy in 2Gy per fraction with weekly 5FU and cisplatin on weeks 1 and 5 vs gemcitabine alone. A large field was treated to 60Gy without a cone-down. This was combined with previously untested in the concurrent setting dose-intensified chemotherapy. Not surprisingly, only 42% of patients were able to receive 75% or more of the planned concurrent radiation and chemotherapy dose compared to 73% in the chemotherapy alone group. Both groups continued to receive gemcitabine thereafter until toxicity or progression. Median OS was better in the gemcitabine alone arm (13 vs. 8.6 months, *p* = 0.03) undermining the role for RT in the management of LAPC in the era of gemcitabine. It should be noted that the particularly intensive CRT regimen that resulted in poor compliance made the interpretation difficult. The other recent trial to compare chemoradiation to chemotherapy alone was conducted by the Eastern Cooperative Oncology Group (ECOG 4201). This trial compared gemcitabine-based chemoradiation to a total dose of 50.4Gy in 28 fractions followed by weekly gemcitabine to gemcitabine alone. Fields were reduced after 39.6 Gy. It randomized 74 patients before being stopped for poor accrual. A modest median survival benefit was seen in the chemoradiation arm (11.1 vs 9.2 months) [8]. Greater grade 4 toxicity was noted in the RT arm, although the combined rates of grade 3–4 toxicity was similar.

**Table 1:** Modern randomized trials of conventionally fractionated radiotherapy

Study	N	Treatment Arms		mOS (months)	2y OS
FFCD-SFRO [5]	119	60Gy/30 fractions + 5Fu + cis- > gem		8.6	~ 15%^a^
		gem		13, *p* = 0.03	~ 21%^a^
ECOG 4201 [8]	74	50.4Gy/28 fractions - > gem		11.1	12%
		gem		9.2, *p* = 0.017	5%
LAP-07 [7]	269^b^	gem+/−erlotinib ->	54Gy + cape	15.2	~ 25%^a^
			gem	16.5, NS	

^a^Estimated from Kaplan-Meier curves

^b^Results from the second randomization are shown (patients who did not progress on induction chemotherapy)

The study with the greatest impact on clinical practice is the LAP 07 trial [7]. After receiving gemcitabine +/− erlotinib for 4 months, 269 patient were randomized to 54Gy in 1.8 Gy per fraction with concurrent capecitabine or to 2 more months of gemcitabine. In, contrast to the trials mentioned above, the fields were limited to gross disease with a margin, without additional prophylactic lymph node coverage. The median overall survival was not improved by addition of chemoradiotherapy (16.5 vs 15.2 months, *p* = 0.083). However, the use of chemoradiotherapy was associated with reduced rates of local disease progression (32% vs 46%, *p* = 0.03), longer interval to re-initiation of therapy (6.1 vs 3.7 months, *p* = 0.02) and a trend toward improved progression-free survival (HR = 0.78, *p* = 0.06). This was achieved with acceptable incremental toxicity, mainly nausea. Collectively these results show that conventionally fractionated chemoradiation up to 60Gy can produce a modest local control benefit, but only minimal, if any, effect on survival. The reason why a local control benefit is not translating into a survival benefit is likely multifactorial, and likely largely influenced by the high metastatic rate seen in this disease. However, another possibility is that for at least the subgroup of patients with predominantly locoregional disease progression, gains in local control have not been significant enough to make a difference in survival. This underscores the need for further dose escalation.

Lack of a substantial benefit, coupled with the introduction of more active systemic regimens such as FOLFIRINOX [9] (5-fluoruracil, oxaliplatin, leucovorin, irinotecan) and gemcitabine and nab-paclitaxel [10], have led to a shift at most academic centers to the much more selective use of consolidative standard dose chemoradiation, preferring more convenient low dose stereotactic body radiotherapy (SBRT).

## Lessons from 1- to 5-fraction stereotactic body radiotherapy experience

Stereotactic body radiotherapy (SBRT) enables highly precise delivery of high doses of radiation to small tumor volumes by using image guidance. Increased precision coupled with evidence that a higher dose per fraction is associated with better local control has led to the emergence of 1- to 5-fraction regimens. Evidence across several tumor types suggests that doses of at least 100Gy BED need to be delivered for an ablative effect or > 90% durable local control. SBRT has gained wide acceptance for targets with little motion uncertainty such as spine or brain tumors. It is also an attractive option for tumors occurring in moving organs with parallel functional subunits, such as the lung or liver whereablation of a small volume of the surrounding normal liver or lung tissue carries no significant clinical consequence. In contrast, ablative doses delivered near an organ with serial functional subunits such as the gastrointestinal tract, are not possible without a risk of affecting organ function. This is particularly relevant for sites where organ motion creates a greater degree of uncertainty about the location of the target and sensitive structures at any given time. For pancreatic tumors, dose delivery is limited by the proximity of radiosensitive GI organs, primarily the duodenum, the jejunum and the stomach, and the uncertainty created by respiratory motion and day-to-day differences in luminal organ shape.

Not surprisingly, early studies using ablative or near-ablative doses in 1–3 fractions were associated with significant early and/or late GI side effects (Table 2). A phase II study evaluated single fraction SBRT of 25Gy (BED 87.5Gy for alpha/beta = 10) in 16 patients treated with gemcitabine for 1 cycle before and until progression thereafter [11]. The dose was prescribed to the planning treatment volume (PTV) with central maximal doses ranging from 32 to 40Gy (BED 134.4 to 200Gy). Treatments were delivered with Cyberknife using Synchrony for tracking throughout the respiratory cycle. One-year freedom from local progression (FFLP) was 100%, but at the expense of late GI toxicity. Seven of 15 patients surviving > 4 months after SBRT (47%) developed grade 2–4 gastric or duodenal complications, including 2 patients (13%) with grade 3–4 events, all occurring 4–10 months after SBRT.

**Table 2:** Representative SBRT studies

Study	N	RT Dose	mOS (months)	GI toxicity (Gr ≥ 2)
Schellenberg et al. [11]	16	25Gy × 1	11.4	Acute 19% Late 47%
Hoyer et al. [12]	22	15Gy × 3	5.7	Acute 79%
Herman et al. [13]	49	6.6Gy × 5	13.9	Acute 2% Late 11%

Another early report of SBRT used 45 Gy in 3 fractions (BED 112.5Gy for alpha/beta = 10) in 22 patients [12]. Here the dose was prescribed to the center, with PTV covered by the 67% isodose line receiving 10Gy × 3. Respiratory motion was managed with abdominal compression.RT was delivered using a standard linear accelerator (LINAC) with bony anatomy used for alignment verification. PTV size was significantly larger than in the single fraction study, although tumor sizes in the two studies were similar. The difference in PTV size was likely at least in part due to the residual motion associated with abdominal compression for motion management. Local control was 57% at 6 months.Both acute and late toxicity was high at 79 and 94% respectively, with 5 of 22 patients with severe gastric or duodenal mucositis or ulceration, including one non-fatal perforation.

Although, comparison of hypofractionated regimens using the linear quadratic model may be somewhat inaccurate, collectively these early experiences showed that dose escalation using very hypofractionated regimens (1–3 fractions) is associated with excess toxicity, which in this setting may be further exacerbated by any set-up uncertainties including residual respiratory motion or using large GTV to PTV expansions to account for such uncertainties.

To ensure safety, clinicians have adopted fractionation schemes of 25–33 Gy in 3 to 5 fractions, which amounts to only 54.78Gy BED using the standard linear-quadratic conversion. A prospective multi-institutional study using 33 Gy in 5 fractions in 49 patients receiving gemcitabine before (up to 3 weeks) and after SBRT, resulted in only minimal acute and late GI toxicity, 2 and 10%, respectively. Unfortunately, 1 year FFLP was only 78% with a median OS of 13.9 months, which is not significantly different from results seen with conventionally fractionated chemoradiation [13]. Our recent retrospective single institution analysis showed similar outcomes with 33Gy in 5 fractions compared to conventional treatments of 50.4-56Gy in 1.8–2.0Gy per fraction [14]. However, a study using the National Cancer Center Database, which included 8450 patients with LAPC showed a modest improvement in OS of 13.9 vs 11.6 months with SBRT, which translated to a more modest absolute benefit at 2 years (21.7% vs 16.5%, *p* = 0.0014), reaching statistical significance due to the large numbers [15]. With the advantage of patient convenience, 5-fraction low dose SBRT is a reasonable community standard. However, it falls short of the goal of durable local tumor control which could translate into a meaningful survival benefit.

## Ablative hypofractionation: moving toward a new standard

Any hope of improving outcomes in LAPC requiresdose escalation beyond 33Gy in 5 fractions. However, safe delivery of higher dose per fraction as a part of a 5-fraction regimen while respecting normal tissue constraints is only possible for a select few patients with tumors far away from the luminal GI tract. Relying on first principles of radiobiology, one way to achieve a higher effective total dose while maintaining an acceptable risk of toxicity is by increasing the number of fractions. Therefore, incorporating the precision of the SBRT technique into a more protracted course is one way to continue dose intensification in LAPC. Fractionation also has the added benefit of “randomizing” the internal day-to-day organ motion, making it less likely that unintended high dose will be delivered to a normal structure that may move closer to the target on any given day.

**Fig. 1:**
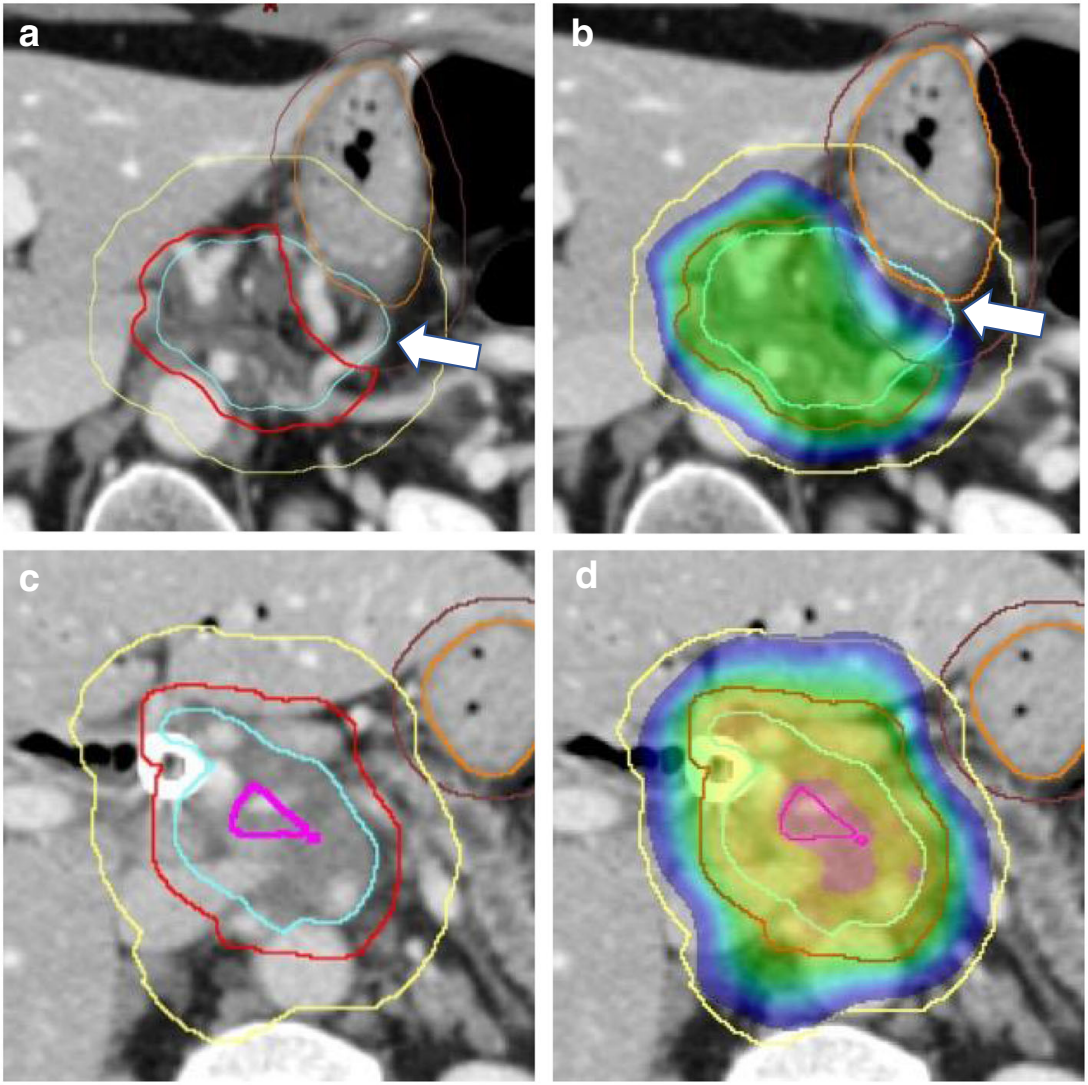
Contouring and plan evaluation. **a** and **c** Simulation CTs showing GTV (cyan), PTV high dose (red) and PTV microscopic dose (yellow) as well as stomach (orange) with a carve-out structure (brown) used to ensure exclusion of stomach from PTV high dose as demonstrated by the white arrow. **b** and **d** Dose distributions with the lowest displayed dose set to the critical max point dose for stomach (60Gy). White arrow indicates that 60Gy isodose line is away from the surface of the stomach, which was achieved by creating a PRV (not shown). **c** and **d** An example that includes an optional PTV ultra-high dose (magenta)

Another conceptual change that would facilitate dose escalation is abandoning the goal of dose homogeneity with the tumor planning treatment volume (PTV). Dose homogeneity as a planning goal is largely a carry-over from more conventional planning approaches. Whenlarge treatment fields contained the target as well as the organs at risk, hotspots within the irradiatedvolume were undesirable. However, when the planning treatment volume is small and limited to the tumor, excluding all sensitive normal structures, a hotspot within that treatment volume has no detrimental effect. On the contrary, allowing a hotspot may improve the conformality of the high dose distribution thereby enabling dose escalation. Importantly, the center of a tumor is typically more hypoxic than the periphery and, therefore, more radioresistant. A hotspot within a more radioresistant portion of the tumor will only be of benefit. Thus our novel treatment planning strategy represents a three-part approach of (1) covering as much of the tumor as possible with an ablative dose while (2) placing supra-ablative hotspots in the center and (3) restricting the areas directly abutting the GI tract to safe doses used in conventional radiotherapy (Fig. 1).

The original series combining these concepts with the stereotactic techniques prescribed 63–70 Gy in 28 fractions or 67.5 Gy in 15 fractions; BED, 77.2–97.9 Gy. Moderate inspiration breath hold respiratory gating with daily breath hold CT-on-Rails fiducial free 3D image registration were used for motion management and image guidance. Unprecedented 3-year OS of 35% and 5-year OS of 18% far exceed historical controls with < 5% in unresected patients surviving past 5 years [16]. These results compare favorably to surgical resection in patients with less advanced local disease and constitute a proof of principle that definitive radiation doses can result in a meaningful long-term survival. An ongoing phase II clinical trial (NCT03523312) is evaluating these doses in a prospective manner.

Daily adaptive planning using novel MR linear accelerators is another way to overcome the limitations posed by the motion of the GI tract. This technology allows visualization of the tumor and critical GI structures, as well as the ability to create a plan of the day that maximizes dose to the pancreas and conforms it to the GI organs at risk on a fraction-by-fraction basis [17] This technique, Stereotactic MR-guided Adaptive Radiation Therapy [18] was utilized to deliver ablative doses of radiation (67.5 Gy / 15 fractions; 50 Gy / 5 fractions) based on the original promising results from MD Anderson listed above [16]. When these were compared with patients receiving non-ablative doses of MRI-guided radiation, there was a significant survival advantage with overall survival from diagnosis of 71% at 2 years in the SMART patients and 25% in the standard dose patients [19]. Moreover, the SMART patients had no grade 3 or higher toxicity, whilst 3 patients in the standard, non-adaptive group had grade 3 or higher toxicity. A prospective, phase II multi-institutional study (NCT03621644) investigating 50 Gy in 5 fractions with SMART is open, and we will await these results to see if they show similar effectiveness.

This technology provides a useful platform to dose escalate pancreatic tumors without increased fractionation. The challenges from a population-based perspective are the limited availability of the technology, and labor intensiveness of the workflow requiring physician/physicist time at each fraction. For patients and institutions that do not have access to this technology, optimized cone beam image-guidance delivered in 15 to 25 fractions with adaptive planning on as-needed basis is a more workflow-friendly approach.

## Practical considerations

### Ablative hypofractionation technique using cone beam image-guidance

Our current approach is to use IMRT with a simultaneously integrated boost (SIB) dose painting, typically with 2 or 3 different planning target volumes (PTVs) (a microscopic dose, a SIB to the GTV, and if possible, a second SIB to a higher dose to the hypoxic center). We predominantly use 15 or 25-fraction schedules based on the proximity to the luminal GI tract. For tumors located within 1 cm of the GI tract, we use a 25-fraction regimen, for tumors more than 1 cm away we use a 15-fraction regimen, with a 5-fraction regimen reserved for very select patients with no nearby GI structures. Typical regimens are listed in Table 3. The bowel dose constraints are based on a previous analysis and listed in Table 3 [20]. With these constraints, no grade 4 or greater bleeding events have occurred to date.

**Table 3:** Ablative radiotherapy prescription definitions and normal tissue constraints

	Planning Volumes	Definition	Doses by Fractionation Scheme	
			15-Fraction	25-Fraction
Prescriptions	Microscopic Extension PTV	CTV + 5 mm CTV = GTV + 1 cm + CA, SMA, +/− porta hepatis, +/− splenic hilum basins	37.5Gy/15	45Gy/25
	High Dose PTV	GTV + 0-5 mm margin excluding GI OAR + 5–7 margin	67.5Gy/15	75Gy/25
	Ultra High Dose PTV^a^	1 cm contraction of High Dose PTV	90Gy/15	100Gy/25
Constraints	Stomach-Duodenum PRV	Stomach and duodenum segments 1 and 2 + 3-5 mm	Dmax <45Gy	Dmax <60Gy
	Small Bowel PRV	All other small bowel + 3-5 mm	Dmax <40Gy	Dmax <55Gy
	Large Bowel PRV	Large bowel + 3-5 mm	Dmax <50Gy	Dmax <65Gy

^a^Possible in select patients only

#### Simulation

We simulate patients in the supine position with customized immobilization and arms elevated. Using the Varian RPM system, we obtain deep inspiration breath hold (DIBH) scans with diagnostic CT pancreatic protocol (150 mL iodinated contrast at 5 mL/s) with imaging at 45 s after the start of contrast administration and a second image obtained at between 1 min 30 s and 2 min after the start of the contrast bolus. This technique allows maximal contrast enhancement of the surrounding parenchyma around the tumor as well as arterial and portal venous enhancement on the first scan, which is usually used as the planning CT scan.

#### Contouring

The most critical aspect of contouring is to exclude all organs at risk (OARs) with an additional safety margin from the high and ultra-high dose PTVs (Fig. 1, Table 3). For this we contour three GI organs at risk that have distinct constraints during the planning process, (1) stomach with the first two segments of the duodenum, (2) the rest of small bowel and (3) large bowel. A margin of 3 to 5 mm is added to create the corresponding planning OAR volumes (PRVs) to be used as avoidance structures during planning. To create PTV _high dose_, a margin of 0-5 mm is added to the gross tumor volume (GTV) of the primary tumor, then all organs at risk (OARs) with an additional safety margin of 5 to 7 mm are excluded. The exact margin depends on the length of the interface of the tumor with the OAR, with greater margins utilized for cases with more extensive abutment. In this process, the edge of the PTV _high dose_ will be separated from the PRVs by at least 2 mm, effectively preventing the high dose gradient from falling immediately adjacent to the sensitive organ where small amount of uncertainty can place the organ at risk within a very high dose region (Fig. 1b, d). To create a PTV_microscopic dose_, we first create a CTV by expanding the GTV of the primary tumor and involved nodes by 1 cm and including the celiac axis and superior mesenteric artery nodal basins in the CTV. and then adding a 5 mm set-up uncertainty margin. In the appropriate clinical context, porta hepatis and splenic hilum nodal basins may also be included. In select cases, where the tumor is large enough and the GTV does not involve bile ducts or the aorta, a PTV_very high dose_ is created by doing a contraction of the PTV_high dose_ by 1 cm.

#### The dual purpose of DIBH

Management of both respiratory and day-to-day internal organ motion is paramount. At MSK we currently use DIBH respiratory gating using RPM Varian system with daily DIBH cone-beam CT (CBCT) image registration. The presence of fiducial markers or a metal biliary stent are required. DIBH is the solution for intra-fraction motion, and enables acquisition of high quality daily CBCT scans by eliminating motion artifact. CBCTs are used to verify the target position as well as day-to-day variation in the position of the adjacent luminal GI tract. The latter is used to select cases for adaptive planning as described below.

It should be noted that despite using DIBH, some CBCTs will provide poor visualization of the luminal GI tract due to other sources of artifact such as luminal gas and peristalsis. However, the additional advantage of a multifraction regimen is that a small number of fractions where the doses to the GI tract are uncertain do not alter the risk profile of the overall treatment plan. Thus, such scans will not necessarily cause treatment delays as long as the position of the target can be verified. The process of using CBCTs to evaluate GI organ position is described in the subsequent section.

Other methods of motion management and image guidance may be used depending on the availability of technology and proficiency of a particular center. These include gating, tumor tracking or abdominal compression for respiratory motion management. In addition to CBCTs, CT-on-Rails or MRI may be used for image guidance.. For all methods of image guidance, motion management will reduce artifact.

#### Selective adaptive planning

**Fig. 2:**
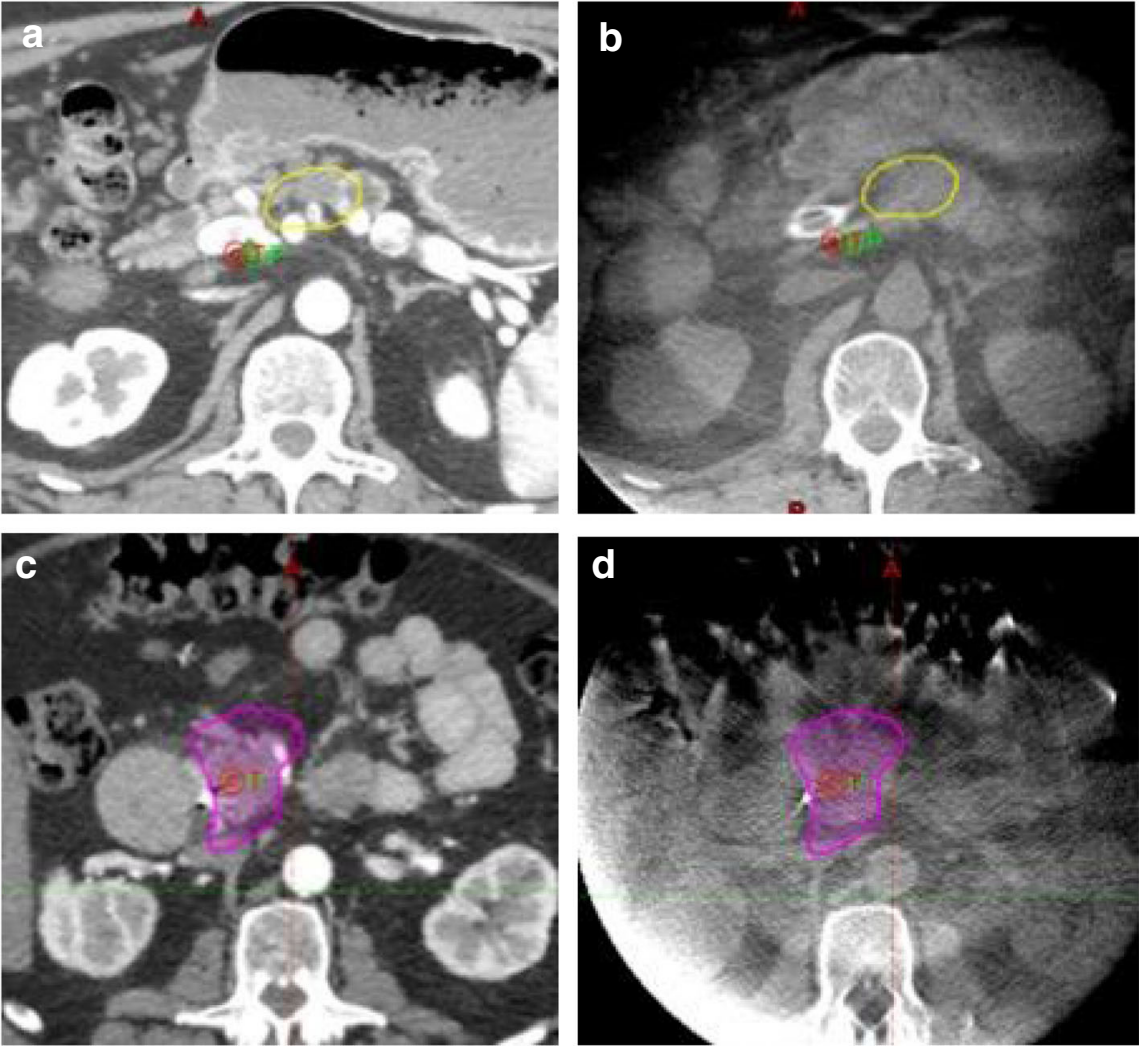
CBCTs are used verify the target position as well as day-to-day variation in the position of the adjacent luminal GI tract. Simulation CTs of two patients displaying the critical max point dose for stomach (yellow) (**a**) and small bowel (magenta) (**c**). Corresponding DIBH CBCT images displaying the same isodose lines (**b** and **d**) are shown to the right. Stomach position may be affected by filling with food and air (**a** and **b**), while the duodenum is very reproducible (**c** and **d**)

Adaptive planning is used as a solution for non-random motion of the GI tract. We evaluate day-to-day organ motion using daily CBCT by projecting maximum point dose isodose line (Table 3) as a structure on our daily CBCTs (Fig. 2). The position of the stomach, duodenum, jejunum and colon with respect to the projected IDLs is easily noted. Adaptive planning is triggered when the same part of an organ crosses that isodose line more than one third of the time. Such selective adaptive planninghas been borne out of experience of the past 10 years. This approach minimizes the number of adaptive plans, and increases operational efficiency. The most common reasons for adaptive planning are related to gas in the stomach, non-random jejunal motion, and gas in the duodenal bulb. When an adaptive plan is necessary, a CBCT fused to the simulation CT can be used to re-contour the OARs on the simulation CT and re-plan without repeating the simulation.

#### Concurrent chemotherapy

Most patients treated with ablative hypofractionated technique to date have received concurrent radiosensitizing chemotherapy. Current MSKCC standard is oral capecitabine twice daily on the days of radiation. Main toxicities include nausea, fatigue, diarrhea, and hand-foot symptoms. The exact contribution of radiosensitization to ablative radiotherapy is not known, but there are possible advantages to both locoregional and systemic disease control.

### Current techniques with stereotactic MR guided adaptive radiation therapy

#### Important aspects of simulation

Patients are simulated with a guided breath hold, supine, with one arm up or both arms down to ensure comfort during the treatment. IV contrast is used similarly as above.

#### Contouring / GI OARs

The most critical aspect of contouring is to identify the stomach, duodenum, small and large bowel within 3 cm of the CTV. The CTV includes the gross tumor, contoured generously to include the superior mesenteric artery and celiac artery when feasible. The CTV is expanded by 3 mm to create a PTV.

#### The role of MRI motion management

Motion management and accuracy of treatment delivery are important in these high dose treatments. The MRI-linear accelerator used automatically processes 4 cine images per second, allowing the patient to be treated with guided breath holds or with gating on free breathing, based on patient comfort.

#### Daily adaptive planning

Daily adaptive planning is the hallmark of this technique. On each fraction, the patient undergoes a couch shift to align the CTV based on simulation. At this time, the GI OARs are re-contoured within 3 mm of the PTV. A new plan is generated whenever the volume of each GI OAR exceeds 1 cc above 33 Gy for a 5-fraction treatment. Each fraction is evaluated separately, since technology does not yet exist to deformably map dose between different bowel loops.

## Conclusions

Treatment paradigms for locally advanced pancreas adenocarcinoma have evolved significantly over the last several years, primarily due to the expanded chemotherapy options. Likewise, there have been significant improvements in radiation therapy delivery techniques with the advent of SBRT.However the potential offered by these techniques has not been fully harnessed with the commonly used 1–5 fraction SBRT regimens. These technological advances enable delivery of radiotherapy doses that are at least 1.5 as potent as conventionally fractionated schedules or the commonly used low dose 1–5 fraction SBRT regimens, and are predicted to be ablative. Multiple single institution series show promising early results, and there are ongoing phase II studies investigating ablative radiation using a CBCT- and an MR-based approaches.

## Data Availability

Not applicable.

## Abbreviations

BED: Biologically effective dose
CBCT: Cone beam CT
CTV: Clinical treatment volume
DIBH: Deep inspiration breath hold
ECOG: Eastern Cooperative Oncology Group
FFCD-SFRO: Fédération Francophone de Cancérologie Digestive and Société Française de Radiothérapie Oncologique
FFLP: Freedom from local progression
FOLFIRINO×: 5-fluoruracil, oxaliplatin, leucovorin, irinotecan
GTV: Gross tumor volume
LAPC: Locally advanced unresectable pancreatic cancer
LINAC: Linear accelerator
OAR: Organ at risk
OS: Overall survival
PRV: Planning organs at risk volumes
PTV: Planning treatment volume
SBRT: Stereotactic body radiation
SIB: Simultaneously integrated boost
SMART: Stereotactic MR-guided Adaptive Radiation Therapy
